# Mcl-1 protects eosinophils from apoptosis and exacerbates allergic airway inflammation

**DOI:** 10.1136/thoraxjnl-2019-213204

**Published:** 2020-04-17

**Authors:** Jennifer M Felton, David A Dorward, Jennifer A Cartwright, Philippe MD Potey, Calum T Robb, Jingang Gui, Ruth W Craig, Jürgen Schwarze, Christopher Haslett, Rodger Duffin, Ian Dransfield, Christopher D Lucas, Adriano G Rossi

**Affiliations:** 1 University of Edinburgh Centre for Inflammation Research, Queen's Medical Research Institute, Edinburgh BioQuarter, UK; 2 Division of Allergy and Immunology, Cincinnati Children’s Hospital Medical Centre, Cincinnati, Ohio, USA; 3 Department of Pharmacology and Toxicology, Dartmouth College Geisel School of Medicine, Hanover, New Hampshire, USA

**Keywords:** eosinophil biology, allergic lung disease, asthma mechanisms

## Abstract

Eosinophils are key effector cells in allergic diseases. Here we investigated Mcl-1 (an anti-apoptotic protein) in experimental allergic airway inflammation using transgenic overexpressing human Mcl-1 mice (hMcl-1) and reducing Mcl-1 by a cyclin-dependent kinase inhibitor. Overexpression of Mcl-1 exacerbated allergic airway inflammation, with increased bronchoalveolar lavage fluid cellularity, eosinophil numbers and total protein, and an increase in airway mucus production. Eosinophil apoptosis was suppressed by Mcl-1 overexpression, with this resistance to apoptosis attenuated by cyclin-dependent kinase inhibition which also rescued Mcl-1-exacerbated allergic airway inflammation. We propose that targeting Mcl-1 may be beneficial in treatment of allergic airway disease.

## Introduction

Eosinophils are key immune cells in the pathogenesis and propagation of allergic airway diseases, including eosinophilic asthma.^1–3^ The restoration of tissue homoeostasis following inflammation requires the cessation of proinflammatory signalling and removal of recruited immune cells.^4^ Resolution of eosinophilic inflammation can be achieved through a combination of inflammatory cell apoptosis and subsequent phagocytic removal (efferocytosis), via transepithelial migration and mucociliary clearance, or via alternative forms of death including necrosis.^5^ Notably, reduced eosinophil apoptosis and defective efferocytosis are both reported in asthmatic patients, and are associated with increasing disease severity. These observations suggest the importance of these mechanisms in the resolution of allergic disease.

We have shown that downregulation of Mcl-1 in human eosinophils occurs concurrent with induction of apoptosis following treatment with cyclin-dependent kinase inhibitor (CDKi) drugs, and that induction of eosinophil apoptosis attenuates allergic lung inflammation in mice in vivo.^6^ In addition, eosinophil Mcl-1 can be dynamically regulated in response to proinflammatory cytokines. Together, these findings suggest that Mcl-1 may be a potential target for ameliorating allergic disease. Despite this, the direct effects of Mcl-1 on allergic airway inflammation in vivo have not been determined. Here we investigated the impact of manipulating Mcl-1, using both pharmacological and genetic approaches, on eosinophil viability and allergic airway inflammation.

## Methods

In vivo experiments were performed under the UK Home Office Animals (Scientific Procedures) Act 1986, following approval by local ethics committee. Female mice (8 to 16 week; wild type (WT) (Charles River or littermate controls)) and transgenic mice overexpressing human Mcl-1 (hMcl-1)^7^ on a C57Bl6 background underwent ovalbumin (OVA)-induced allergic airway inflammation, with bronchoalveolar lavage fluid (BALF) and tissue acquired and processed as described.^1 6^ AT7519 (30 mg/kg in sterile saline) was administered as a single intraperitoneal dose. Mice were housed in a specific-pathogen-free facility with standard husbandry. Mice were not randomised but allocated on the basis of genotype, with a single animal serving as the experimental unit, with all treatments and assessments performed in the morning. BALF total cell counts were measured by NucleoCounter with BALF cell composition analysed by flow cytometry. Interstitial cells were analysed following collagenase digest of lungs after bronchoalveolar lavage and perfusion with phosphate-buffered saline. Lung histology was blinded prior to analysis by a lung pathologist. Cytokines were analysed by ELISA (BALF) or Luminex (lung homogenate). For ex vivo culture of BALF eosinophils (CD45^+ve^/CD11c^-ve^/Ly6G^-ve^/Siglec-F^+ve^), BALF cells were incubated (5×10^6^/mL) in serum-free Iscove’s Modified Dulbecco’s Medium (IMDM) for 48 hours +/-AT7519, Q-VD-OPh as per figure legends.

Mouse bone marrow-derived eosinophils (bmEos) were generated from WT and hMcl-1 mice as described.^1^ Light microscopy or flow cytometric analysis of Annexin-V/propidium iodide (PI) or DAPI staining was used to identify viable, apoptotic and necrotic bmEos. Western blotting was performed as described.^1 8 9^

Data expressed as mean±SEM, analysed using GraphPad Prism and FlowJo software. Significance accepted at p<0.05.

## Results

### Mcl-1 overexpression exacerbates allergic airway inflammation in vivo

**Figure 1:**
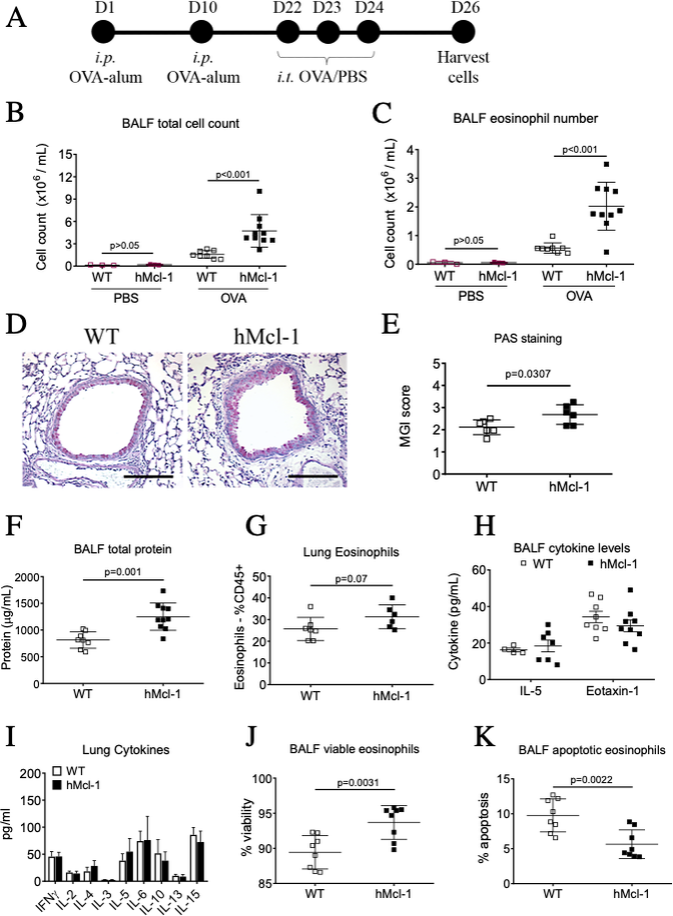
Overexpression of Mcl-1 exacerbates allergic airway inflammation in vivo. (A) Schema of experimental protocol, (B) total bronchoalveolar lavage fluid (BALF) cell counts assessed by NucleoCounter and (C) BALF eosinophil number analysed by flow cytometry (CD45^+^/CD11c^-^/Siglec-F^+^ Singlets) from PBS or OVA-challenged mice at day 26 (n=3 for PBS treated groups, n=8–10 for OVA groups). (D) Representative lung sections stained with periodic acid-Schiff (PAS) from WT and hMcl-1 mice (x200 original magnification, scale bar=150 µm) with (E) quantification of mucus production as assessed by the mucus-goblet index (MGI) (n=6 both groups). (F) BALF total protein (n=8 WT, n=10 hMcl-1), (G) lung interstitial eosinophils (as % of CD45^+^ events; n=7 WT, n=6 hMcl-1), (H) BALF IL-5 & eotaxin-1 cytokine levels (eotaxin n=8 WT, n=9 hMcl-1, IL-5; n=4 WT, n=7 (hMcl-1), and (I) lung cytokines (n=7 WT, n=6 hMcl-1). The percentage of (J) viable (annexin V^-ve^/PI^-ve^ cells) and (K) apoptotic (annexin V^+ve^/PI^-ve^) BALF eosinophils (n=8 WT, n=8 hMcl-1), assessed by flow cytometry. Data analysed by one-way analysis of variance with a Newman-Keuls multiple-comparisons test (B and C), or by Student’s t-test (E–I). hMcl-1, human Mcl-1; ip, intraperitoneal; ns=non significant; OVA, ovalbumin; PBS, phosphate-buffered saline; WT, wild type.

To investigate Mcl-1 in allergic airway inflammation, WT or hMcl-1 mice were sensitised and challenged with OVA (or PBS as control)^6^ (figure 1A). Western blotting confirmed expression of human Mcl-1 in bmEos) from hMcl-1 mice (online supplementary figure 1). No change in BALF total cell counts or eosinophil numbers (online supplementary figure 2) were observed in hMcl-1 transgenic mice following PBS challenge (figure 1B, C). However, hMcl-1 mice had an exacerbated allergic response to OVA with increased BALF cellularity, eosinophil numbers, airway mucus production and BALF total protein (figure 1B–F), with an increase in the proportion of interstitial eosinophils that did not reach statistical significance (figure 1G). The proportion of lung alveolar macrophages, neutrophils and T cell subsets were similar between WT and hMcl-1 mice following OVA (online supplementary figure 3). Despite increased eosinophils in hMcl-1 mice, BALF eotaxin (CCL11) and IL-5 (key cytokines for eosinophil recruitment and lifespan) were unaltered (figure 1H), with multiple cytokines from homogenised lung showing similar levels between WT and hMcl-1 mice (although with a trend towards increased IL-4 in the hMcl-1 mice; 18.2±2.9 vs 28.4±4.0 pg/mL p=0.06, figure 1I) . However, the proportion of viable eosinophils (Annexin V^-ve^/PI^-ve^) was increased in hMcl-1 mice, with a concurrent reduction in apoptotic eosinophils (Annexin V^+ve^/PI^-ve^; figure 1J, K).

10.1136/thoraxjnl-2019-213204.supp1 Supplementary data

### Mcl-1 overexpression delays eosinophil death

**Figure 2:**
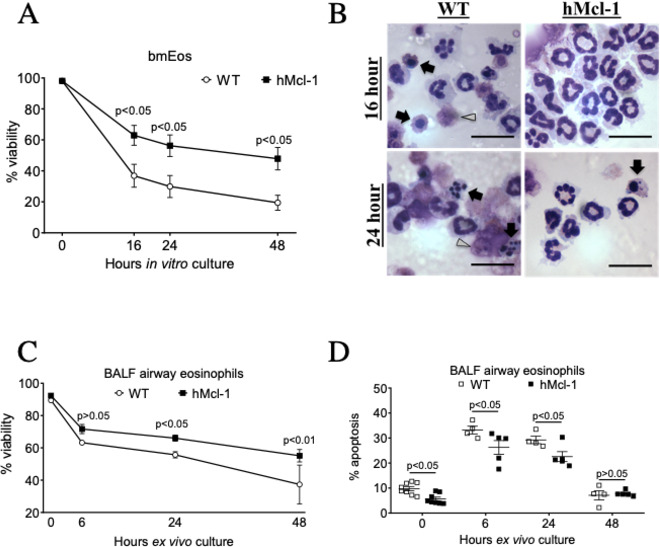
Overexpression of Mcl-1 delays bone marrow-derived eosinophil (bmEos) and airway eosinophil apoptosis. (A and B) WT and hMcl-1 transgenic bmEos were cultured over 48 hours with assessment of eosinophil viability and apoptosis by flow cytometry (annexin V/propidium iodide binding) (n=5 WT, n=4 hMcl-1). (B) Representative cytocentrifuge preparations (×1000 original magnification, scale bar=20 µm) at 16 and 24 hours for WT and hMcl-1 bmEos. *Black arrows* point to bmEos with apoptotic morphology, *Grey arrowheads* indicate cellular fragmentation of bmEos. (C and D) Airway eosinophils from WT and hMcl-1 OVA-challenged mice were cultured over 48 hours prior to assessment of (C) viability and (D) apoptosis by flow cytometry (annexin V/DAPI binding) (n=8 WT, n=8 hMcl-1 at 0 hour; n=4 WT, n=5 hMcl-1 at 6 hour, 24 hours & 48 hours). Data are expressed as mean±SEM as analysed by two-way analysis of variance with a Bonferroni test, p values as indicated. BALF, bronchoalveolar lavage fluid; hMcl-1, human Mcl-1; OVA, ovalbumin; WT, wild type.

We next investigated whether enhanced eosinophil viability in the hMcl-1 mice in vivo was intrinsic to eosinophils or a secondary phenomenon due to potential alterations in the inflammatory milieu. Serum and IL-5-starvation of bmEos demonstrated that hMcl-1 bmEos had enhanced viability (figure 2A, B). Similarly, airway eosinophils from OVA-challenged hMcl-1 mice demonstrated enhanced survival (figure 2C) and reduced apoptosis (figure 2D) during ex vivo culture.

### The cyclin-dependent kinase inhibitor AT7519 reverses delayed eosinophil apoptosis in hMcl-1 mice to rescue allergic inflammation

**Figure 3:**
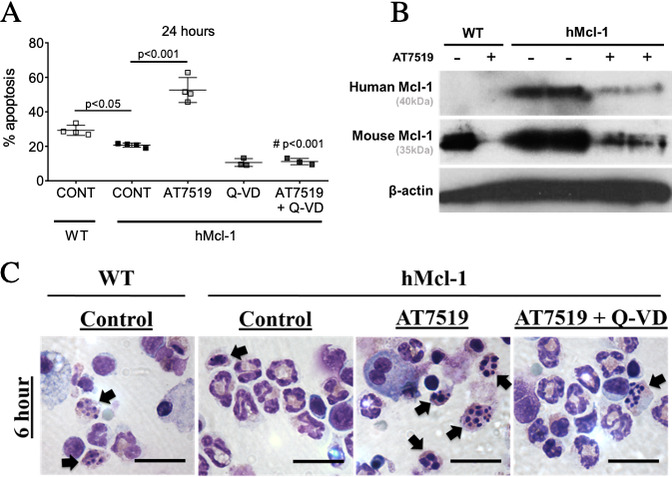
AT7519 reverses delayed eosinophil apoptosis. (A) Eosinophil apoptosis (AnnV^+ve^/Ly6G^−ve^/Siglec-F^+ve^) from WT and hMcl-1 mice at 24 hours with AT7519 (1 µM) with or without the caspase inhibitor Q-VD (10 µM) as assessed by flow cytometry (n=3–4). (B) Bronchoalveolar lavage cells from WT or hMcl-1 OVA-challenged mice treated +/-AT7519 (1 µM) for 4 hours prior to lysing and Western blotting for hMcl-1 (40KD), mouse Mcl-1 (35kD) and β-actin (42kD). (C) Representative cytocentrifuge preparations (×1000 original magnification, scale bar=20 µm) at 6 hours for WT and hMcl-1 control cells and hMcl-1 cells treated with AT7519 (1 µM) and combined AT7519 (1 µM) and Q-VD (10 µM). *Black arrows* indicate apoptotic eosinophils. Data expressed as mean±SEM, analysed by one-way analysis of variance with a Newman-Keuls multiple-comparisons test, ^###^p< 0.001 versus AT7519-treated group or as indicated (A). hMcl-1, human Mcl-1; OVA, ovalbumin; WT, wild type.

**Figure 4:**
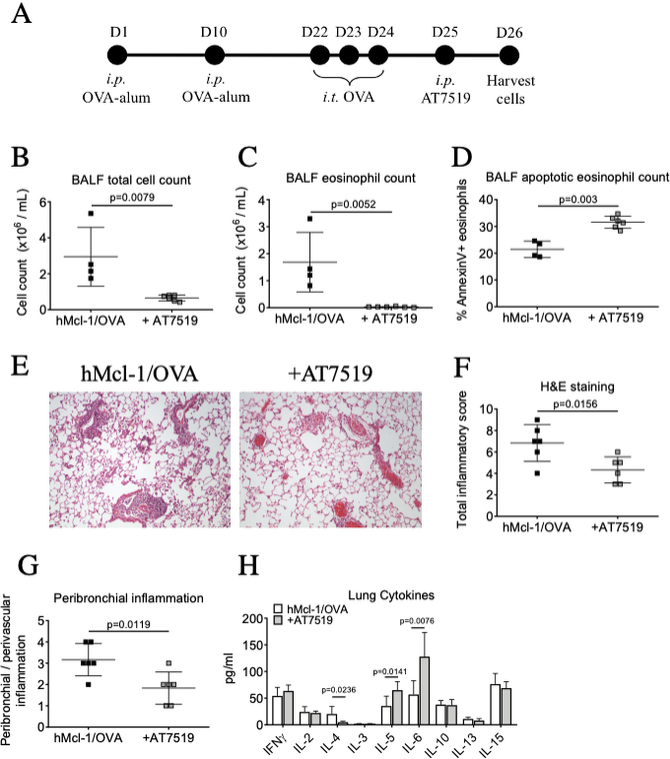
AT7519 reverses delayed eosinophil apoptosis in hMcl-1 mice to rescue allergic inflammation in vivo. (A) Schema of experimental protocol. (B) Total bronchoalveolar lavage fluid (BALF) cell counts from OVA-challenged hMcl-1 mice after 26 days with either PBS or AT7519 treatment on day 25. (C) BALF eosinophil number and (D) percentage of BALF apoptotic eosinophils (AnnV^+ve^/DAPI^-ve^, CD45^+ve^/CD11c^-ve^/Ly6G^-ve^/Siglec-F^+ve^ cells). (B-D n=4 hMcl-1, n=6 +AT7519). (E) Representative lung sections stained with H&E (X100 original magnification) with (F) quantification of total inflammation and (G) peribronchial/perivascular inflammation. (H) Lung cytokines analysed by Luminex. (F-H n=6 hMcl-1, n=6 +AT7519). Data expressed as mean±SEM, analysed by Student’s t-test. hMcl-1, human Mcl-1; OVA, ovalbumin; PBS, phosphate-buffered saline.

The CDKi AT7519 increased apoptosis of airway eosinophils from hMcl-1 mice. This AT7519-induced apoptosis was caspase-dependent (inhibited by the broad-spectrum caspase inhibitor Q-VD) and was associated with reduced Mcl-1 expression (figure 3A–C). To assess the therapeutic potential of AT7519 to attenuate Mcl-1-exacerbated allergic airway inflammation in vivo, AT7519 was administered 24 hours after intratracheal ovalbumin (figure 4A). This significantly reduced BALF cellularity and eosinophils, with increased Annexin V^+ve^ eosinophils (figure 4B–D). H&E sections demonstrated a reduction in the number of perivascular and peribronchial eosinophils and lymphocytes following treatment with AT7519 (figure 4E–G) while no difference was observed in bronchial mucin production by PAS staining (data not shown). Analysis of lung cytokines demonstrated a reduction in IL-4 and increases in IL-5 and IL-6 (figure 4H). Overall, these data confirmed that AT7519 rescues Mcl-1-exacerbated allergic airway inflammation in vivo.

## Discussion

Understanding mechanisms and regulatory processes controlling inflammation resolution is the focus of recent intense investigation.^4 10^ Mechanisms can be tissue and scenario dependent, but apoptosis and subsequent efferocytosis of inflammatory cells are recognised as major processes ensuring inflammation resolution and tissue repair. Here we determined the role of Mcl-1 in both the regulation of eosinophil lifespan and in driving allergic airway inflammation.

We report several novel findings that extend our understanding of eosinophil apoptosis in controlling inflammation and tissue injury in airway allergy. First, we show that overexpression of Mcl-1 leads to exacerbated allergic airway inflammation as determined by several inflammation parameters including BALF cellularity, eosinophil numbers, BALF protein and airway mucus production. Whether manipulation of Mcl-1 also leads to changes in airway hyperresponsiveness or remodelling remains to be determined, but is a logical future extension of our current work.

Second, we show that Mcl-1 overexpression reduces eosinophil death both in vitro and in vivo in the context of airway allergy, extending our previous observations that Mcl-1 is a key regulator of neutrophil apoptosis,^9 11^ and that CDKi induce eosinophil apoptosis concurrent with Mcl-1 loss.^12^ While cytokine-mediated changes or maintenance in eosinophil Mcl-1 (by IFN-γ and IL-5) have previously been reported, this is to our knowledge the first report that Mcl-1 aggravates allergic airway inflammation in vivo. While we cannot exclude the possibility that the regulatory role of Mcl-1 on inflammation in vivo involves other cell types, we have previously shown that modulation of eosinophil apoptosis or apoptotic eosinophil clearance is sufficient to alter allergic airway inflammation.^1 6^

Finally, we show that the apoptosis protection conferred to eosinophils by Mcl-1 overexpression could be overcome by pharmacological lowering of Mcl-1 levels using indirect Mcl-1 inhibition with a CDKi. Furthermore, Mcl-1-exacerbated allergic inflammation in vivo could be rescued by the same approach suggesting that Mcl-1 targeting approaches are capable of overriding the complex pro-survival signals that exist in the inflamed lung. Of note, this is in contrast to glucocorticoid-induced eosinophil apoptosis which can be abrogated by the presence of cytokines such as IL-5.

In summary, our results demonstrate Mcl-1 as a significant regulator of eosinophil longevity and the outcome of allergic airway inflammation in vivo. We propose that manipulation of Mcl-1 could be exploited for the treatment of allergic diseases such as eosinophilic asthma in humans.
