# Supplementary figures and images for: Mcl-1 protects eosinophils from apoptosis and exacerbates allergic airway inflammation
Source: Thorax. 2020 Apr 17;75(7):600–5. doi: 10.1136/thoraxjnl-2019-213204 (PMC7361019; doi:10.1136/thoraxjnl-2019-213204)

Figure S1

Felton JM *et al.*

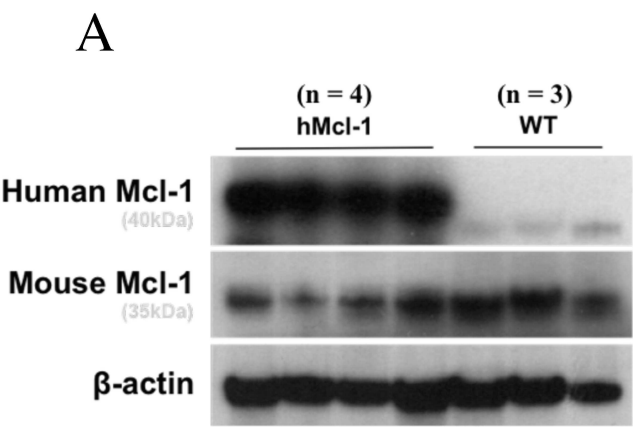

Figure S2

Felton JM *et al.*

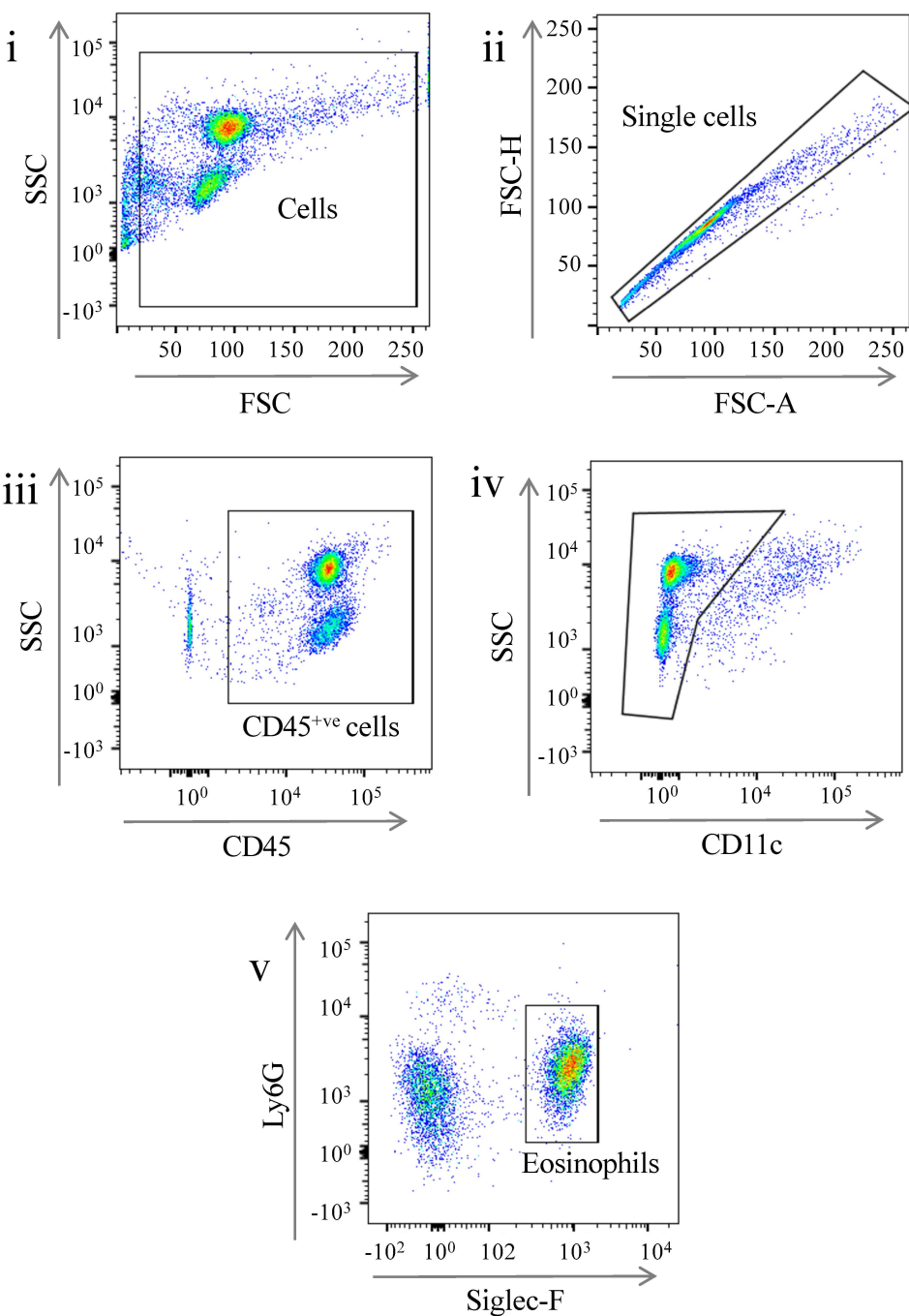

Figure S3

Felton JM *et al.*

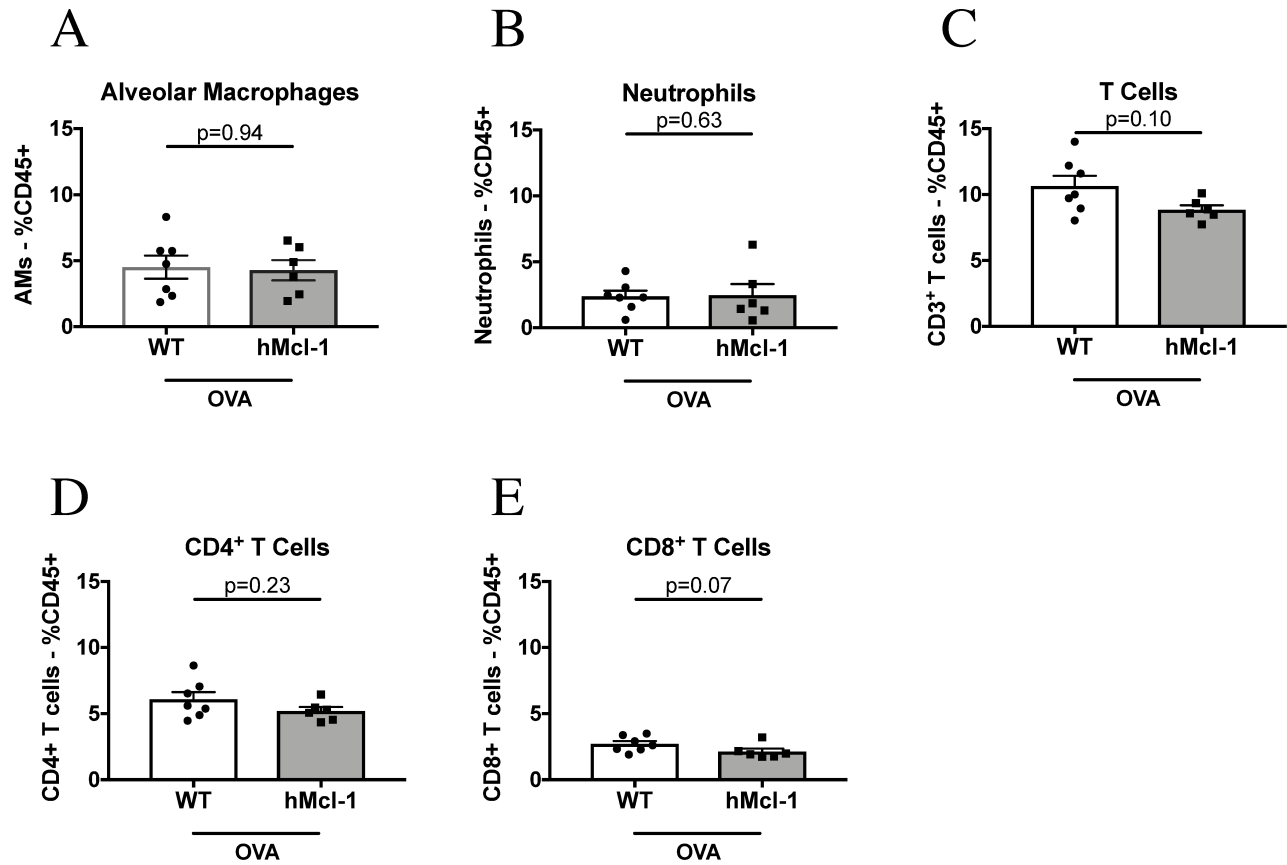

Supplement: Supplementary data [file thoraxjnl-2019-213204supp001.pdf]
